# Impact of asymmetric tethering on outcomes after edge-to-edge mitral valve repair for secondary mitral regurgitation

**DOI:** 10.1007/s00392-021-01961-5

**Published:** 2021-11-16

**Authors:** Lukas Stolz, Mathias Orban, Daniel Braun, Philipp Doldi, Martin Orban, Konstantin Stark, Michael Mehr, Julius Steffen, Kornelia Löw, Christian Hagl, Steffen Massberg, Michael Näbauer, Jörg Hausleiter

**Affiliations:** 1 Medizinische Klinik und Poliklinik I, Klinikum der Universität München, Marchioninistraße 15, 81377 Munich, Germany; 2 German Center for Cardiovascular Research (DZHK), Partner Site Munich Heart Alliance, Munich, Germany; 3 Herzchirurgische Klinik und Poliklinik, Klinikum der Universität München, Munich, Germany

**Keywords:** MitraClip, Heart failure, Secondary mitral regurgitation, Transcatheter mitral valve edge-to-edge repair, Asymmetric tethering

## Abstract

**Background:**

The impact of postero-anterior and medio-lateral mitral valve (MV) tethering patterns on outcomes in patients undergoing transcatheter edge-to-edge repair (M-TEER) for secondary mitral regurgitation (SMR) is unknown.

**Methods:**

The ratio of the posterior to anterior MV leaflet angle (PLA/ALA) in MV segment 2 was defined as postero-anterior tethering asymmetry. Medio-lateral tethering asymmetry was assessed as the ratio of the medial (segment 3) to lateral (segment 1) MV tenting area. We used receiver-operating characteristics and a Cox regression model to identify cut-off values of asymmetric anteroposterior and medio-lateral tethering for prediction of 2 year all-cause mortality after TMVR.

**Results:**

Among 178 SMR patients, postero-anterior tethering was asymmetric in 67 patients (37.9%, PLA/ALA ratio > 1.54). Asymmetric medio-lateral tethering (tenting area ratio > 1.49) was observed in 49 patients (27.5%). M-TEER reduced MR to ≤ 2 + in 92.1% of patients; MR reduction was less effective in the presence of asymmetric postero-anterior tethering (*p* = 0.02). A multivariable Cox regression model identified both types of asymmetric MV tethering to be associated with increased all-cause 2-year mortality (postero-anterior tethering asymmetry: HR = 2.77, CI 1.43–5.38; medio-lateral tethering asymmetry: HR = 2.90, CI 1.54–5.45; *p* < 0.01).

**Conclusions:**

Asymmetric postero-anterior and medio-lateral MV tethering patterns are associated with increased 2-year mortality in patients undergoing M-TEER for SMR. A detailed echocardiographic analysis of MV anatomy may help to identify patients who profit most from M-TEER.

**Graphical abstract:**

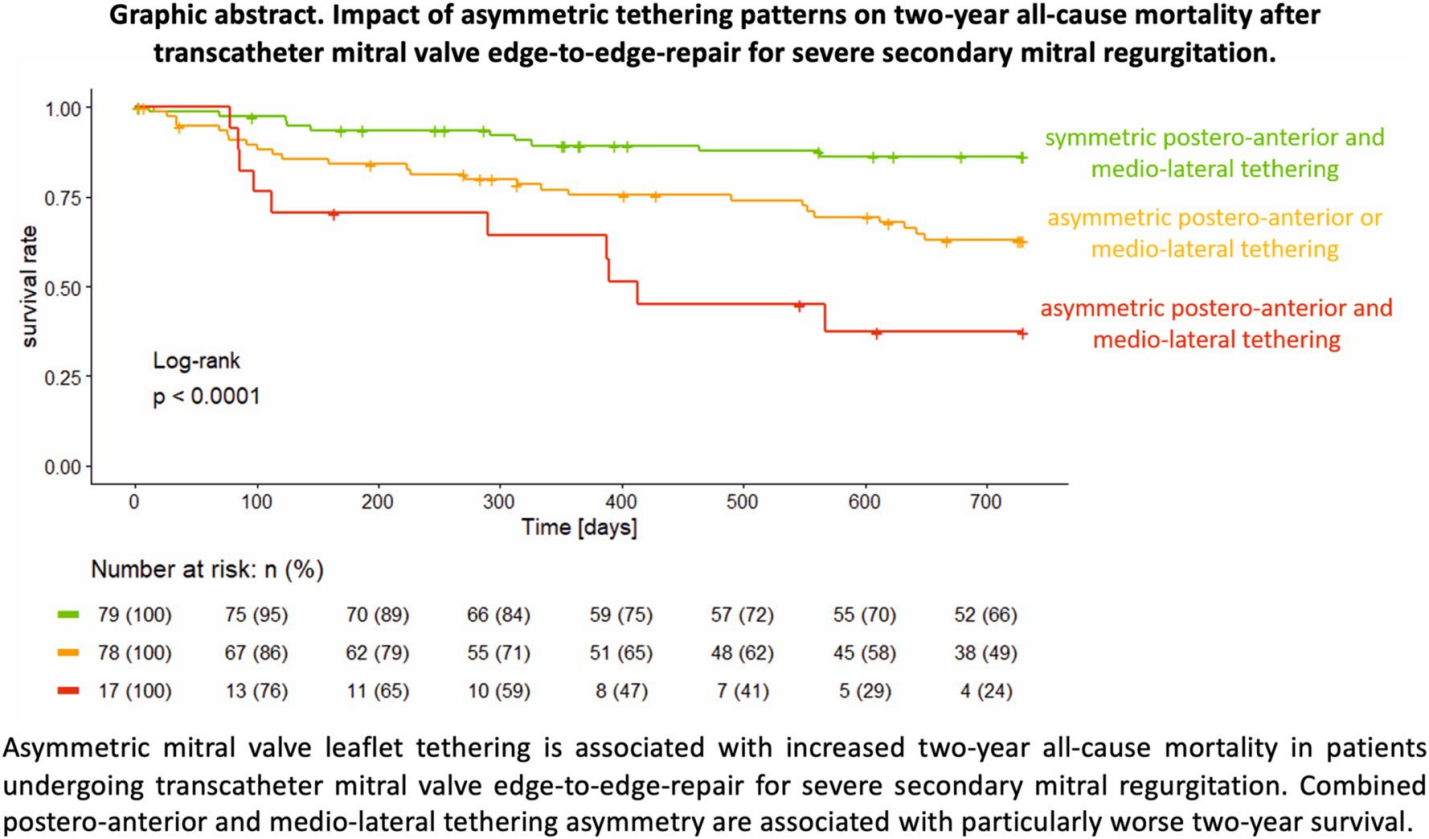

**Supplementary Information:**

The online version contains supplementary material available at 10.1007/s00392-021-01961-5.

## Introduction

Severe secondary mitral regurgitation (SMR) has a poor prognosis and causes substantial morbidity in patients with heart failure and reduced ejection fraction (HFrEF) [1]. Therefore, mitral transcatheter valve edge-to-edge repair (M-TEER) is a guideline-recommended therapy in symptomatic high-risk patients with severe SMR after application of guideline-directed medical treatment and, if indicated, cardiac resynchronization therapy [2]. Two recent randomized-controlled trials (COAPT and MITRA-FR) showed conflicting results regarding hospitalization for heart failure and mortality [3, 4]. The results of both randomized studies and other registries stress the importance of meticulous preprocedural patient selection for M-TEER to achieve best possible results and long-lasting benefit [5, 6].

In HFrEF patients, left-ventricular ejection fraction (LV-EF) progressively deteriorates and left heart geometry alters due to ventricular, atrial, and annular dilation [7, 8]. Ventricular remodelling particularly affects the geometric configuration of the subvalvular mitral apparatus including papillary muscles, as the latter become displaced [9–11]. This leads to restriction of MV leaflets with consecutive MR defined as type IIIb in the Carpentier classification. The underlying pathology resulting in SMR is heterogeneous with ischemic cardiomyopathies resulting in ischemic SMR, dilated cardiomyopathies resulting in non-ischemic SMR, and finally atrial fibrillation and heart failure with preserved ejection fraction resulting in atrial SMR (ASMR) [12–14]. Thus, atrioventricular remodelling itself is a rather heterogeneous process and may lead to different patterns of MV leaflet tethering. In the current literature, the term asymmetric MV leaflet tethering is applied heterogeneously [15–17]. Some authors use it to describe a disproportionate restriction of the posterior, compared to the anterior MV leaflet motion [16]. Other authors use this term to describe a more severe tethering in medial segment 3 (S3) of the MV, compared to central S2 and lateral S1 as represented by tenting area and volume in the respective segments. Medio-lateral tethering asymmetry is believed to be a consequence of inferoposterior myocardial infarction (MI) with subsequent apical displacement of the medial papillary muscle [15]. Data about the impact of both postero-anterior and medio-lateral asymmetric MV tethering patterns on outcomes after M-TEER for severe SMR are absent. Only restricted posterior MV leaflet tethering has been reported to predict persistence or recurrence of MR severity > 2 + up to 12 months after intervention [18].

In patients undergoing MV surgery for SMR, asymmetric leaflet tethering patterns have been shown to be associated with increased rates of residual MR and worse long-term outcome [19, 20]. Since M-TEER is now the interventional therapeutic option of choice in SMR [2], the aim of this study was to evaluate the prognostic impact of asymmetric postero-anterior and medio-lateral leaflet tethering in M-TEER-treated SMR patients regarding procedural MR reduction, symptomatic improvement, and 2-year all-cause mortality.

## Methods

### Patient selection and treatment process

We included consecutive patients with moderate-to-severe (3+) or severe (4+) SMR undergoing M-TEER between July 2013 and March 2019 using MitraClip NT, NTR or XTR (Abbott, Santa Clara, California, USA) at our center. Due to significant changes of MV leaflet anatomy, patients with prior surgical or transcatheter mitral valve repair have been excluded from the analysis, as well as interventions being performed as bridge to heart transplantation or interventions with concomitant tricuspid transcatheter valve repair. Furthermore, patients without a detailed preprocedural echocardiographic assessment of the MV pathology were excluded from this study. After transthoracic (TTE) and transoesophageal echocardiographic (TEE) assessment, all patients were discussed by an interdisciplinary heart team and deemed at high or prohibitive surgical risk. Follow-up included survival status and New York Heart Association (NYHA) function class. Patients unable to attend follow-up examinations at our center were interviewed per telephone or seen by their local practitioners.

### Echocardiography and endpoints

Echocardiographic evaluation of the cardiac chambers and MV was performed according to the recommendations of the European Association of Cardiovascular Imaging (EACVI) [21]. Detailed MV anatomy was assessed retrospectively by TEE image analysis. Papillary muscle distance and medio-lateral MV annular diameter were derived from a mid-oesophageal two-chamber view. Two-dimensional cross-sectional long-axis views were used for measurement of the following MV parameters in each MV segment: anterior (ALA) and posterior (PLA) leaflet angles were defined as angles between the mitral annular plane and tangent on the root of the anterior or posterior MV leaflet (Supplementary Fig. 1). Tenting height was measured as the distance between perpendicular lines through mitral annular plane reaching to the most ventricular located coaptation point of the MV leaflets. In the same frame, tenting area and postero-anterior MV annular diameter were assessed as shown in Supplementary Fig. 1. All anatomic MV measurements were performed for each MV segment in end-systole.

Effective regurgitant orifice area (EROA) and regurgitant volume (RegVol) were measured by the proximal isovelocity surface area method. The LV sphericity index was defined as ratio of LV length and width in an end-diastolic apical four-chamber view. LV width was measured as the broadest diameter of the LV in an apical four-chamber view. End-diastolic (LV-EDV) and end-systolic left-ventricular volume (LV-ESV) were measured using apical two- and four-chamber view according to Simpson’s biplane summation of disc method. Left-ventricular end-diastolic and systolic diameters, as well as MR vena contracta (MRVC) were obtained in a parasternal long-axis view. Systolic pulmonary artery pressure was estimated by addition of maximum systolic tricuspid valve pressure gradient with estimated right atrial pressure derived from inferior vena cava width. Grading of SMR severity was performed using a comprehensive approach integrating EROA, RegVol, MRVC, and jet morphology based on current guidelines [22, 23]. IntelliSpace Cardiovascular (Version 1.2, Philips Medical Systems, Nederland B.V.) was used for all echocardiographic analyses.

Two-year all-cause mortality was defined as primary endpoint. Postprocedural MR severity was assessed at the end of M-TEER procedure. Symptomatic improvement was evaluated using NYHA functional class.

### Definition of postero-anterior and medio-lateral tethering

**Fig. 1:**
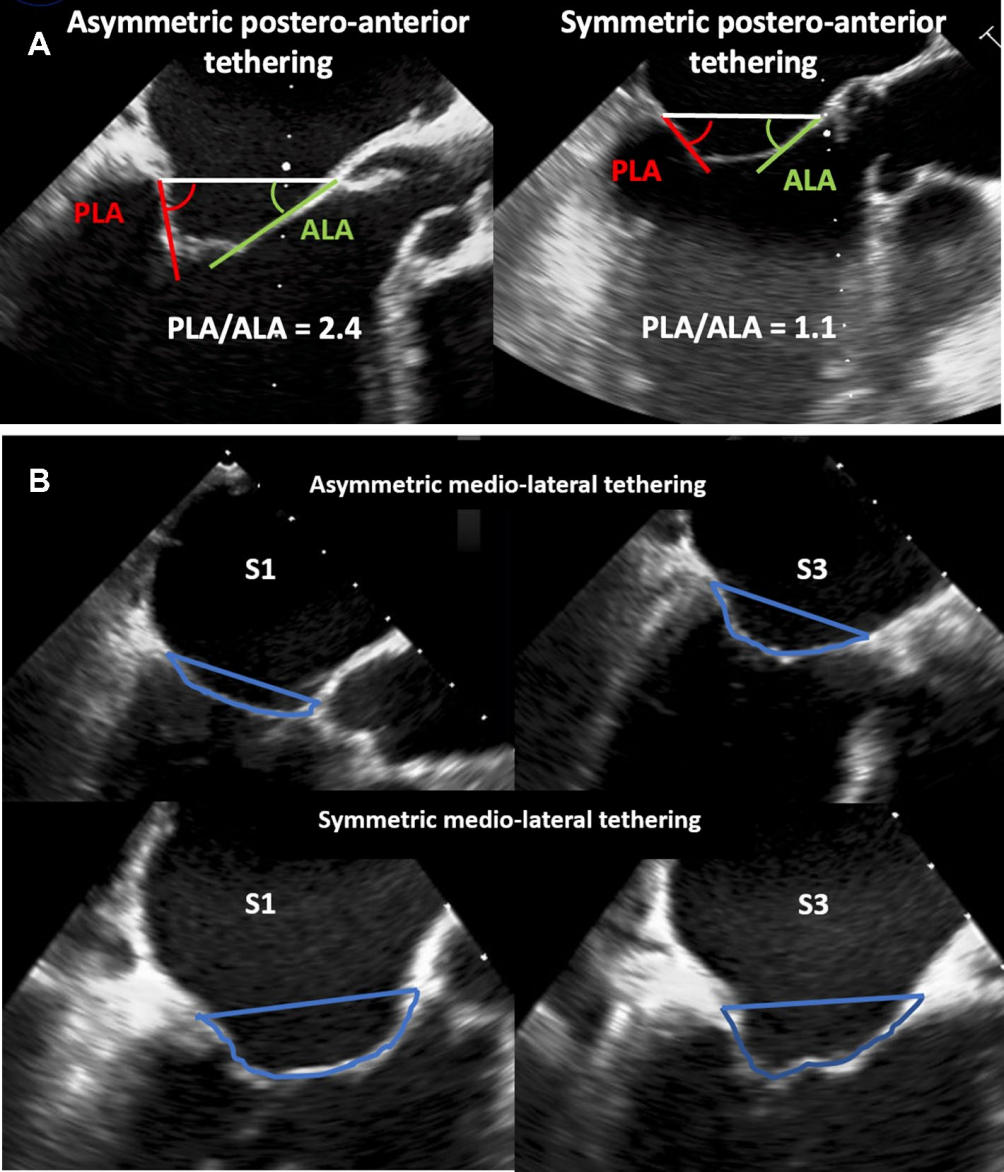
Symmetric and asymmetric postero-anterior and medio-lateral MV leaflet tethering. **A** Transoesophageal echocardiography of a patient with asymmetric postero-anterior tethering (left) and a patient with symmetric postero-anterior leaflet tethering (right). **B** Transoesophageal echocardiography of a patient with asymmetric medio-lateral leaflet tethering (top) and a patient with symmetric medio-lateral leaflet tethering (bottom). *MV* mitral valve; *S* segment

Postero-anterior MV leaflet tethering asymmetry was defined as the PLA/ALA ratio in MV segment 2 (Fig. 1A). Medio-lateral MV leaflet tethering asymmetry was assessed as the ratio of the MV tenting areas between MV segments 3 and 1 (Fig. 1B).

### Statistical analysis

Normality of data was assessed using Kolmogorov–Smirnov and Shapiro–Wilk test. Mann–Whitney *U* or Pearson’s Chi-square test were used for inter-group comparison, as appropriate. The development of NYHA functional class and MR severity after M-TEER for each patient was analysed using the Wilcoxon test. We used receiver-operating characteristics (ROC) analysis and Youden’s J to identify the best discriminatory cut-off values for postero-anterior and medio-lateral asymmetric leaflet tethering in terms of 2-year all-cause mortality and residual postprocedural MR. Multivariable cox analysis and logistic regression included all parameters presenting with *p* < 0.05 in univariable statistics**.** The relationship of tethering symmetry and logarithmic hazard ratio was visualized using spline curves. Kaplan–Meier curves were used for showing survival after M-TEER. The difference of impact of tethering asymmetry on survival was calculated using the log-rank test. Results are displayed as hazard ratio (HR) with 95% confidence interval (CI) and *p* value. A *p* value of < 0.05 was defined as statistically significant. Statistical analyses were performed with SPSS (version 25, IBM, USA) and R (R version 4.0.4).

## Results

### Baseline characteristics and outcome

Overall, 178 patients undergoing M-TEER for moderate-to-severe or severe SMR and sufficient echocardiographic MV assessment (long-axis cross-sectional views of all three MV segments) before the procedure were included. Patients mean age was 72 ± 11 years. The etiology of SMR was ischemic in 106 patients (59.2%). Mean LV-EF was impaired with 35.3 ± 11.2%. All patients had MR grade 3 + (59.6%) or 4 + (40.4%). LV-EDV and LV-ESV were 178.9 ± 69.9 ml and 117.0 ± 57.8 ml, respectively. Most patients presented highly symptomatic in NYHA functional class III (74.2%) or IV (25.7%). Average renal function was impaired in the majority of patients (estimated glomerular filtration rate [eGFR] 53.6 ± 22.8 ml/min). Clinical and echocardiographic baseline characteristics are shown in Table 1 and Table 2, respectively. Patients were treated using MitraClip NT (*n* = 148, 83.1%), NTR (*n* = 14, 7.9%), or XTR (*n* = 15, 8.4%). One patient (0.6%) received both, MitraClip NTR and XTR.

**Table 1:** Clinical baseline characteristics

Parameter	Baseline	*n*
Age, years	71.6 ± 11.0	178
Male sex	109 (60.9)	178
Previous MI	61 (34.1)	178
Previous CABG	25 (14.0)	178
Previous stroke or TIA	18 (10.1)	178
ICD/CRT/PM	88 (49.2)	178
Extracardiac arteriopathy	26 (14.5)	178
Atrial fibrillation or flutter	121 (67.6)	178
BMI, kg/m^2^	25.4 ± 4.5	178
EuroSCORE II, %	8.3 ± 9.2	158
Coronary artery disease	106 (59.2)	178
NYHA functional class		
II	1 (0.6)	178
III	132 (74.2)	
IV	45 (25.3)	
eGFR, ml/min	53.6 ± 22.8	175
Previous TAVR or SAVR	19 (10.6)	178
Medication		
ACE/AT inhibitors	123 (74.1)	165
ß blocker	140 (84.3)	165
Calcium antagonists	12 (7.3)	164
Statins	93 (56.7)	163
ASA	94 (56.6)	165
Diuretics	154 (86.0)	165
Aldosterone antagonists	85 (53.1)	159

Data are presented as mean ± standard deviation or number (%)

*ACE* angiotensin conversion enzyme; *ASA* acetylsalicylic acid; *AT* angiotensin; *BMI* body mass index; *CABG* coronary artery bypass graft; *CRT* cardiac resynchronization therapy; *eGFR*  estimated glomerular filtration rate; *HTX* heart transplantation; *ICD* implantable cardioverter-defibrillator; *MI* myocardial infarction; *NYHA* New York Heart Association; *SAVR* surgical aortic valve repair; *TAVR*  transcatheter aortic valve repair; *TIA* transient ischemic attack

**Table 2:** Echocardiographic baseline characteristics

Parameter	Baseline	*n*
MR severity		
3 +	106 (59.6)	178
4 +	72 (40.4)	
MR severity post		
1 +	117 (65.7)	178
2 +	47 (26.4)	
3 +	12 (6.7)	
4 +	2 (1.1)	
Number of implanted devices		
0	10 (5.6)	178
1	80 (44.9)	
2	82 (46.1)	
3	5 (2.1)	
4	1 (0.6)	
TR severity		
0 +	6 (3.4)	177
1 +	83 (46.9)	
2 +	64 (36.0)	
3 +	23 (12.9)	
4 +	1 (0.6)	
MR EROA PISA, cm^2^	0.26 ± 0.15	174
MR RegVol PISA, ml	37.4 ± 19.3	174
MR vena contracta, cm	0.69 ± 0.22	177
LV-EDV, ml	178.9 ± 69.9	167
LV-ESV, ml	117.0 ± 57.8	167
LV-EDD, mm	61.1 ± 10.5	162
LV-ESD, mm	52.3 ± 10.6	162
LV-EF, %	35.3 ± 11.2	172
LV length, mm	83.8 ± 12.3	171
LV width, mm	57.3 ± 11.0	171
LV sphericity	1.5 ± 0.2	171
LA volume, ml	113.6 ± 53.4	169
Papillary muscle distance, mm	28.7 ± 6.8	139
ML MV annular diameter, mm	38.7 ± 5.3	178
AP MV annular diameter, mm	41.3 ± 6.5	178
MV annular sphericity	0.96 ± 0.36	178
Anterior MV leaflet angle, °	33.8 ± 11.1	178
Posterior MV leaflet angle, °	48.2 ± 33.6	178
AML leaflet length, mm	34.0 ± 6.5	178
PML leaflet length, mm	16.5 ± 4.4	178
Tenting height MV, mm	8.6 ± 3.0	178
Tenting area MV segment 1, mm^2^	185 ± 102	178
Tenting area MV segment 2, mm^2^	270 ± 113	178
Tenting area MV segment 3, mm^2^	182 ± 92	178
Postero-anterior tethering asymmetry	1.54 ± 1.22	178
Medio-lateral tethering asymmetry	1.30 ± 1.33	178
MV mean PG, mmHg	1.7 ± 0.8	176
TAPSE, mm	17.7 ± 4.6	176
TR max PG, mmHg	37.1 ± 12.7	156
sPAP, mmHg	44.4 ± 14.5	98

Data are presented as mean ± standard deviation or number (%)

Unless otherwise specified anatomic measurements refer to MV segment 2

*ALA* anterior mitral valve leaflet angle; *AML* anterior mitral valve leaflet angle; *AP* postero-anterior *EROA* effective regurgitant orifice area; *LA* Left atrium; *LV* left ventricle; *LV-EDD* left-ventricular end-diastolic dimension; *LV-EDV* left-ventricular end-systolic volume; *LV-EF* left-ventricular ejection fraction; *LV-ESD* left-ventricular end-systolic dimension; *LV-ESV* left-ventricular end-diastolic volume; *ML*  medio-lateral; *MR* mitral regurgitation; *MV* mitral valve; *PG* pressure gradient; *PISA* proximal isovelocity surface area; *PLA* posterior mitral valve leaflet angle; *PML * posterior mitral valve leaflet angle; *sPAP* systolic pulmonary artery pressure; *RegVol* regurgitant volume; *TAPSE* tricuspid annular plane systolic excursion; *TR* tricuspid regurgitation

M-TEER led to a procedural decrease of MR severity to grade ≤ 1 + in 117 (65.7%) patients, and ≤ 2 + in 164 (92.1%) patients. Postprocedural MR was 3+ and 4+ in 12 patients (6.7%) and 2 patients (1.1%), respectively. Clinical follow-up rates in eligible patients were 88.8% at 1 year and 82.1% at 2 years. Overall survival rates were 80.4% and 70.6% at 1- and 2-year follow-up, respectively. NYHA class follow-up was available in 121 patients (70.0%).

### Asymmetry of mitral valve leaflet tethering

#### Asymmetric postero-anterior MV tethering

**Fig. 2:**
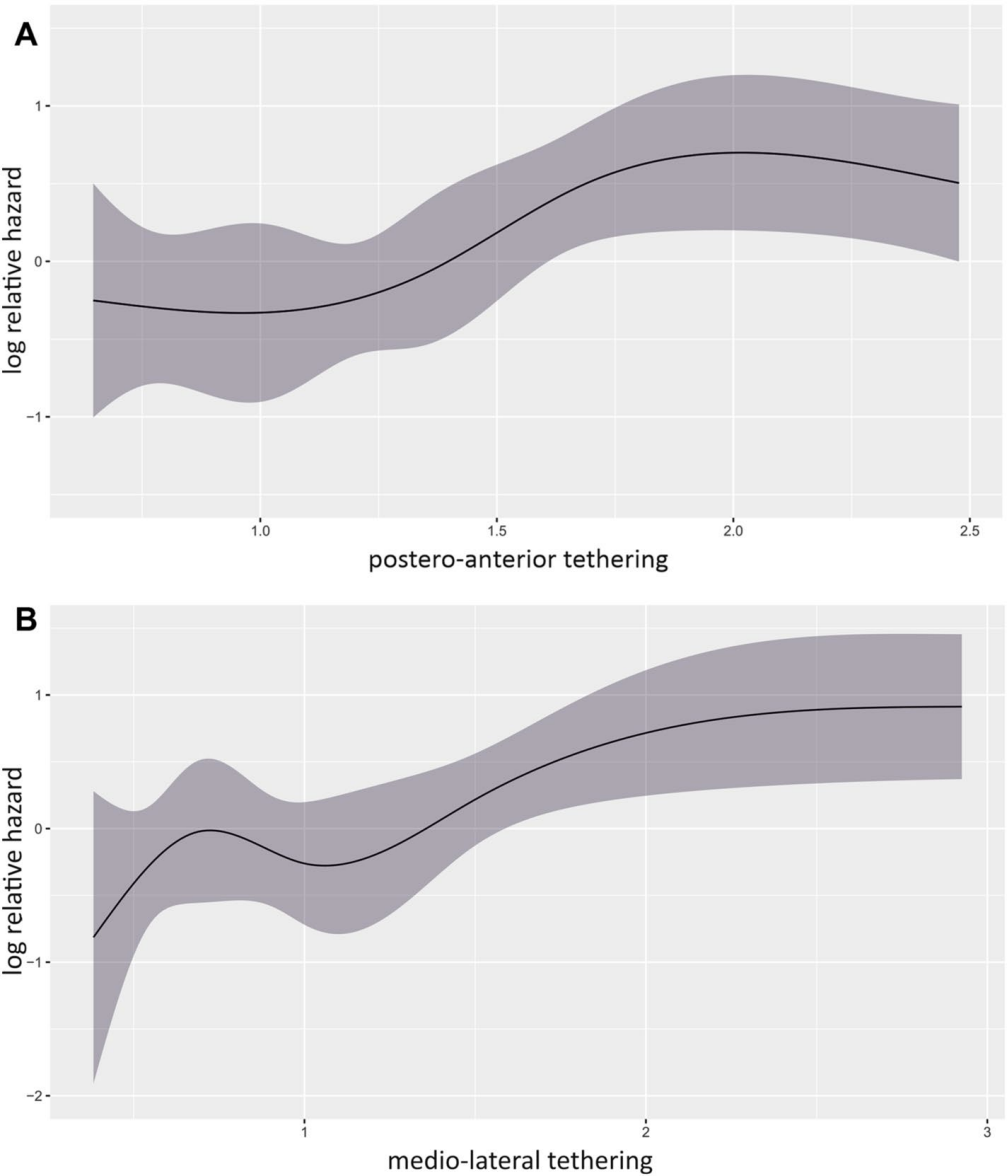
Cox regression spline curves for postero-anterior and medio-lateral tethering asymmetry. **A** Spline curve for postero-anterior tethering asymmetry. **B** Spline curve for medio-lateral tethering asymmetry. Postero-anterior and medio-lateral tethering asymmetry are represented by the PLA/ALA and S3/S1 tenting area ratios, respectively

Table 2 gives a detailed overview on the MV anatomy of the study cohort. Postero-anterior MV leaflet tethering asymmetry was defined as the PLA/ALA ratio in MV segment 2, as tethering was most pronounced within the central MV segment (Supplementary Table 1). Mean postero-anterior tethering asymmetry was 1.54 ± 1.22. The postero-anterior asymmetry was mainly driven by PML restriction (PLA: 48.2 ± 33.6°). ROC analysis revealed that postero-anterior S2 tethering {area under curve [AUC] = 0.61, CI 0.51–0.70, *p* = 0.03} provides discriminatory power regarding prediction of 2-year all-cause mortality. Subsequent calculation of Youden’s J identified a PLA/ALA ratio > 1.54 as best discriminatory value for asymmetric postero-anterior tethering regarding 2-year survival prognosis (sensitivity 0.53; specificity 0.68). Figure 2A graphically outlines the relationship of postero-anterior tethering symmetry and mortality risk after M-TEER. Within the range of PLA/ALA ratio 1.0:2.0, HR had a linear association, while PLA/ALA ratios < 1.0 and > 1.75 had a static association. Sixty-seven patients (37.6%) were identified to have asymmetric postero-anterior tethering with a PLA/ALA ratio > 1.54.

**Fig. 3:**
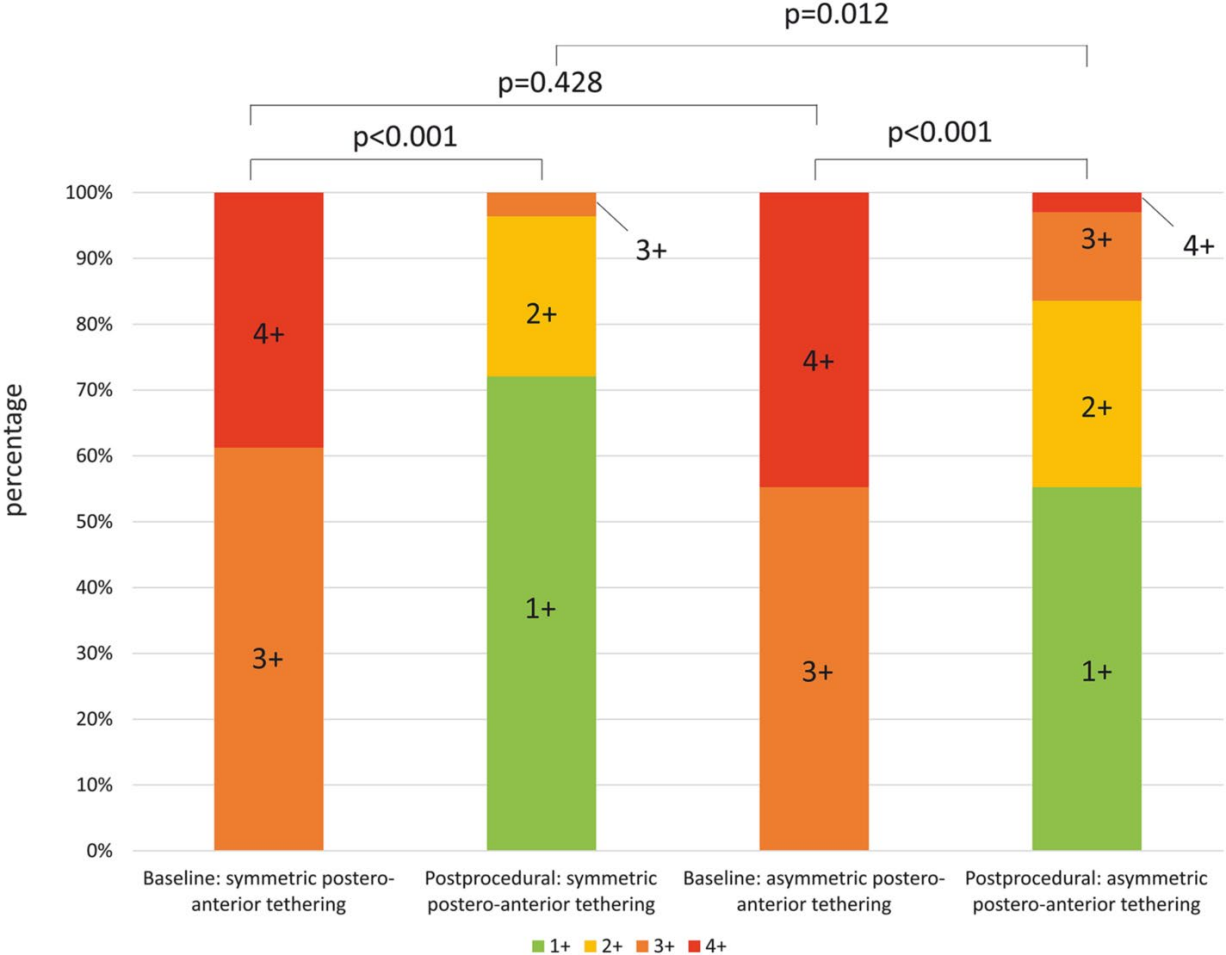
MR reduction in patients with symmetric and asymmetric postero-anterior leaflet tethering. Asymmetric postero-anterior MV tethering is associated with less profound procedural MR reduction in patients undergoing M-TEER for severe MR. *MV* mitral valve; *MR* mitral regurgitation; *M-TEER* transcatheter mitral valve edge-to-edge repair. 178 paired samples

Clinical baseline characteristics did not differ between patients with symmetric and asymmetric postero-anterior tethering, except for a higher prevalence of previous coronary artery bypass graft (CABG) surgery and coronary artery disease (CAD, both *p* = 0.042, Supplementary Table 1) in patients with asymmetric postero-anterior tethering. MR severity expressed by MR EROA, RegVol, and VC was comparable in both groups. A logistic regression model identified CAD {Odds ratio [OR] = 2.19, CI 1.13–4.23, *p* = 0.02} and MV mean PG (OR = 1.53, CI 1.13–4.23, *p* = 0.03) to be associated with asymmetric postero-anterior tethering (Supplementary Table 2). Implantation of at least one clip was successfully performed in 167 patients (93.8%) in the total cohort. Postprocedural MR remained higher in patients with asymmetric postero-anterior tethering (*p* < 0.01), while there was no difference at baseline (Fig. 3, Supplementary Table 3). Among a total of 14 patients with residual MR ≥ 3 + , 10 did not receive any device. The main reason was development of high MV pressure gradients after device positioning (mean gradient before clip retraction: 8.3 ± 1.8 mmHg). In two patients, no device was implanted due to a short, immobile posterior leaflets with extensive asymmetric postero-anterior tethering. Four patients received at least one device. One patient suffered from device detachment of the second implanted clip with the residual MR jet being located near the device. In three patients, residual MR remained ≥ 3 + , after positioning of the first clip. Due to development of high MV pressure gradients after positioning of another clip in the presence of asymmetric tethering, MR could not further be reduced. In these cases, residual MR was located near the already implanted device. Sensitivity analysis identified a PLA/ALA ratio > 1.80 as optimal cut-off for the association with residual postprocedural MR (AUC = 0.69, CI 0.52–0.86, *p* = 0.03).

#### Asymmetric medio-lateral MV tethering

Medio-lateral MV leaflet tethering asymmetry was defined as the ratio of the MV tenting area in MV segment 3 compared to segment 1. ROC analysis and subsequent calculation of Youden’s J revealed an S3/S1 ratio > 1.49 as being associated with survival; thus, we termed this pattern asymmetric medio-lateral tethering (AUC = 0.66, CI 0.56–0.75, *p* < 0.01; sensitivity 0.48, specificity 0.81). Figure 2B depicts the rather cubic than linear relationship of medio-lateral tethering asymmetry and mortality risk after M-TEER. Forty-nine (27.5%) patients presented with asymmetric medio-lateral tethering.

Of note, there were no between-group differences in clinical or echocardiographic parameters at baseline (e.g., MR EROA, RegVol, VC), except for a higher degree of TR severity in patients with asymmetric medio-lateral tethering (Supplementary Tables 1 and 3). Correspondingly, sensitivity analysis did not identify a significant cut-off for the association of medio-lateral tethering symmetry and residual MR (AUC = 0.43, CI 0.26–0.60, *p* = 0.39). Furthermore, logistic regression did not identify parameters being independently associated with asymmetric medio-lateral tethering (Supplementary Table 4).

#### Prognostic value of asymmetric postero-anterior and medio-lateral MV tethering

**Fig. 4:**
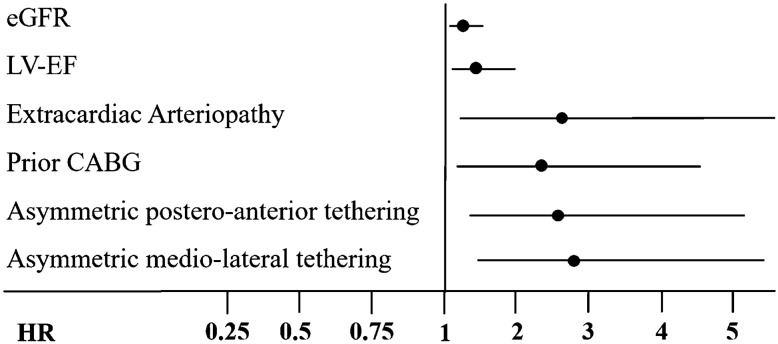
Multivariable Cox model for 2-year all-cause mortality. Predictors for 2-year all-cause mortality after M-TEER for SMR. eGFR per 10 ml/min decrease, LV-EF per 10% decrease; *eGFR* estimated glomerular filtration rate; *LV-EF* left-ventricular ejection fraction; *LV-ESV* left-ventricular end-systolic volume; *HR* hazard ratio; *NYHA* New York Heart Association; *TAPSE* tricuspid annular plane systolic excursion; *M-TEER* transcatheter mitral valve edge-to-edge repair; *SMR* secondary mitral regurgitation, *n* = 164

Predictors for all-cause 2-year mortality in the univariable analysis are summarized in Supplementary Table 5. Multivariable Cox regression revealed LV-EF (per 10% decrease, HR = 1.42, 95% CI 1.04–1.94 *p* = 0.03), eGFR (per 10 ml/min decrease, HR = 1.25, 95% CI 1.06–1.47, *p* = 0.01), prior CABG (HR = 2.30, 95% CI 1.13–4.68, *p* = 0.02), extracardiac arteriopathy (HR = 2.63, 95% CI 1.19–5.78, *p* = 0.02), asymmetric postero-anterior leaflet tethering (HR = 2.77, CI 1.43–5.38, *p* = 0.01), and asymmetric medio-lateral tethering (HR = 2.90, CI 1.54–5.45, *p* < 0.01) to be associated with increased all-cause 2-year mortality (Table 3 and Fig. 4). Patients with concomitant asymmetric postero-anterior and medio-lateral tethering showed particularly reduced 1- and 2-year survival rates compared to those with either asymmetric postero-anterior or medio-lateral tethering or patients without any asymmetric tethering pattern (Graphic abstract).

**Table 3:** COX regression model for all-cause 2-year mortality

	Univariable			Multivariable		
	HR	CI	*p* value	HR	CI	*p* value
Asymmetric medio-lateral tethering	2.609	1.459–4.665	0.001	2.901	1.543–5.451	0.001
Asymmetric postero-anterior tethering	2.332	1.305–4.169	0.004	2.769	1.426–5.376	0.003
LV-EF, per 10% decrease	1.336	1.006–1.775	0.045	1.417	1.037–1.937	0.029
eGFR, 10 ml/min decrease	1.265	1.086–1.478	0.002	1.247	1.058–1.471	0.008
Previous CABG	2.637	1.367–5.089	0.004	2.298	1.129–4.676	0.022
Extracardiac arteriopathy	3.096	1.631–5.874	0.001	2.627	1.194–5.781	0.016

*n* = 164

*LV-EF* left-ventricular ejection fraction; *eGFR* estimated glomerular filtration rate; *CABG* coronary artery bypass graft; *HR* hazard ratio; *CI*  95% confidence interval

**Fig. 5:**
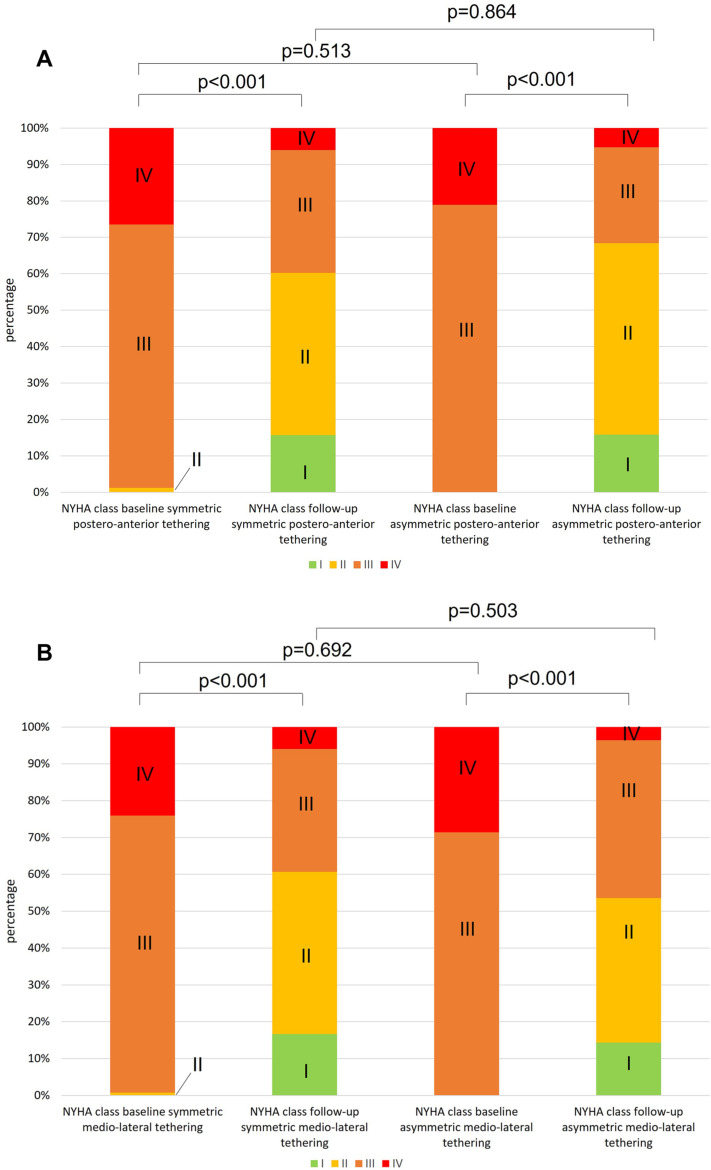
NYHA functional class development. NYHA functional class at baseline and at follow-up examination in patients with symmetric vs asymmetric postero-anterior (**A**) and medio-lateral MV leaflet tethering (**B**). *NYHA* New York Heart Association; *MV* mitral valve. Postero-anterior tethering: 83 paired samples. Medio-lateral tethering: 38 paired samples

NYHA class at baseline did not differ in patients with symmetric vs asymmetric postero-anterior and medio-lateral tethering. We observed NYHA class improvement independent of any leaflet tethering pattern (both *p* < 0.001; Fig. 5A, B).

## Discussion

With more than 10 years of experience, M-TEER is the most widely applied transcatheter treatment approach for high-risk patients with severe SMR. Safety, efficacy, and symptomatic long-term benefit have been shown in several studies [3–5, 24–26]. In this study, we report about the impact of asymmetric MV leaflet tethering on procedural, symptomatic, and prognostic outcomes in patients undergoing M-TEER for severe SMR.

Asymmetric postero-anterior leaflet tethering was present in about 38% of patients. So far, data on the impact of asymmetric tethering patterns on outcomes after M-TEER are sparse. One study identified restricted PML leaflet tethering to be tightly associated with M-TEER failure defined as MR ≥ 3 + 12 months after intervention [18]. Extending these results, we identified asymmetric postero-anterior tethering to be predominantly caused by excessive PML restriction in comparison to moderately impaired AML systolic motion. Consistently, we found postprocedural MR severity to be significantly higher in the presence of asymmetric postero-anterior tethering, despite a comparable baseline MR. Hence, a more eccentric preprocedural MR jet direction may contribute to higher postprocedural MR rates [27].

Asymmetric medio-lateral tethering was associated with substantially elevated mortality rates in this study. In the literature, asymmetric medio-lateral tethering is considered to be a consequence of inferoposterior myocardial infarction and subsequent apical papillary muscle displacement [15].

This is the first study to assess the impact of asymmetric MV leaflet tethering on mortality after M-TEER for SMR. We identified both asymmetric postero-anterior and medio-lateral tethering to be associated with increased all-cause 2-year mortality besides known clinical predictors as left-ventricular and renal function [5]. Patients with a severely distorted valve anatomy comprising concomitant asymmetric postero-anterior and medio-lateral tethering had the worst 2-year survival. Importantly, symptomatic improvement assessed by NYHA functional did not differ between groups of tethering patterns.

We hypothesise that asymmetric postero-anterior MV tethering leads to worse procedural MR reduction and therefore is associated with inferior survival outcome after M-TEER. For asymmetric medio-lateral tethering, the exact pathophysiological link is less obvious. Patients with the latter condition might represent a certain etiological subgroup of SMR patients, who experience unfavourable ventricular remodelling within their underlying cardiomyopathic disease process. Apical displacement of the medial papillary muscle could contribute to this observation, as this unilateral affection of a major subvalvular structure could lead to asymmetric distortion of the MV as expressed by medio-lateral tethering. Nevertheless, further studies (e.g., animal models) are needed to clarify the exact pathophysiologic mechanism.

The current study outlines the importance of asymmetric MV leaflet tethering on both procedural MR reduction and mortality following a transcatheter treatment approach for severe SMR. In MV surgery, both asymmetric postero-anterior and medio-lateral tethering have been identified as conditions associated with higher rates of residual MR, and mortality [19, 20, 28]. Within the past years, novel surgical techniques were developed to tackle this challenge. Subannular repair techniques provide the possibility to reduce the degree of postprocedural tethering and follow-up MR compared to the conventional isolated annuloplasty [29]. Current application of surgical techniques include subannular strategies in addition to undersizing annuloplasty, which suggests a benefit for left-ventricular remodelling and MR recurrence compared to annuloplasty alone [30–32], although this strategy has to be evaluated in randomized trials. All patients included into this analysis were treated using devices without the possibility of independent leaflet grasping. This led to difficulties in grasping the MV leaflets, especially in patients with asymmetric postero-anterior tethering without generating significant MV stenosis. Advanced generations of M-TEER devices (e.g., the PASCAL system [Edwards Lifesciences, Irvine, California] and the fourth-generation MitraClip devices) provide the possibility of independent leaflet grasping [33]. This novel feature might facilitate appropriate leaflet grasping and effective MR reduction even in patients with complicated tethering patterns without generation of significant MV stenosis. Beyond that, the development of transapical or transcatheter MV replacement devices may provide an important treatment alternative for patients with complex MV anatomy. Further studies are needed to optimize the therapeutic pathway for suffering from SMR due to asymmetric MV tethering. A head-to-head comparison of M-TEER vs. surgical MV repair/replacement in HFrEF patients is needed to evaluate the non-inferiority or even superiority between these approaches. This question is currently investigated in the ongoing randomized Mitral Valve Reconstruction For Advanced Insufficiency Of Functional Or Ischemic Origin (MATTERHORN) trial.

## Limitations

This is a retrospective, single-center analysis of M-TEER treated patients without core-lab supervision whose follow-up data were acquired prospectively. Three-dimensional and multiple high-quality biplane two-dimensional imaging in various planes are state-of-the-art echocardiography in evaluating MV anatomy and function, which was necessary to perform the current analyses. Accordingly, a few patients, in whom such detailed imaging was not available, had to be excluded from this analysis. Even after application of a multivariable Cox model, we cannot rule out that further parameters not included into the manuscript may have an impact on the predictive value of asymmetric tethering patterns. Due to the retrospective character of this study, no information on the exact localization of previous MI was available. Since patients with asymmetric tethering had worse survival after M-TEER, there could be bias for NYHA follow-up examination. We further acknowledge that the newly defined cut-offs for the definition of postero-anterior and medio-lateral tethering need further prospective validation in larger M-TEER patient cohorts, especially in the light of possible differences in baseline characteristics between patients.

## Conclusions

Asymmetric tethering of the mitral valve leaflets—either in the postero-anterior or medio-lateral direction—is identified as strong independent predictors for all-cause 2-year mortality in patients with severe SMR undergoing M-TEER. Accordingly, we recommend including the precise evaluation of asymmetric tethering patterns into the routine pre-interventional assessment of SMR to account for potential challenges in MR reduction as well as considering alternative devices including transcatheter mitral valve implantation which may be associated with improved outcomes in such patients. Further randomized-controlled trials are needed to compare different interventional MV repair devices and techniques.

## Supplementary Information

Below is the link to the electronic supplementary material.

Supplementary file1 (DOCX 298 KB)

Supplementary file2 (DOCX 59 KB)

## Data Availability

All authors had unrestricted access to the complete data.
