# Supplementary material for: Impact of asymmetric tethering on outcomes after edge-to-edge mitral valve repair for secondary mitral regurgitation
Source: Clin Res Cardiol. 2021 Nov 16;111(8):869–80. doi: 10.1007/s00392-021-01961-5 (PMC9334427; doi:10.1007/s00392-021-01961-5)
Supplement: Supplementary file 1 — Supplementary file1 (DOCX 298 KB) [file 392_2021_1961_MOESM1_ESM.docx]

**Supplementary Figure 1.** Mitral valve anatomy measurements


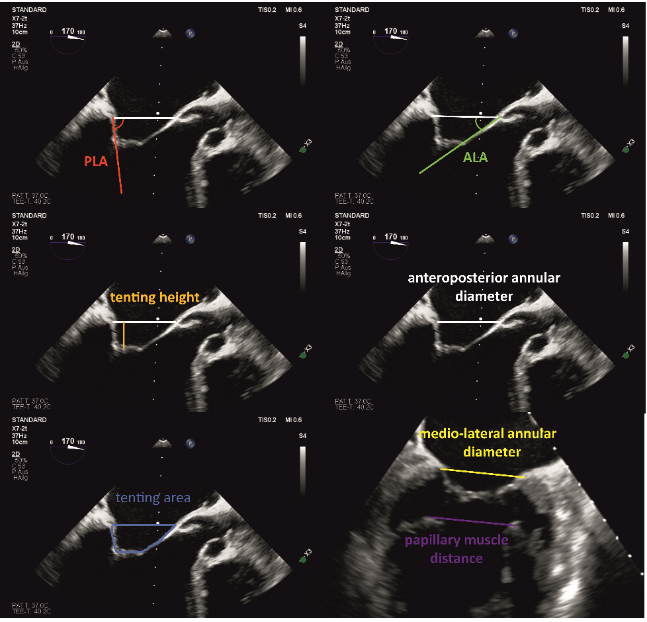


Assessment of mitral valve leaflet angles and tenting height. PLA, ALA, tenting height, postero-anterior annular MV diameter and tenting area were measured in a long axis view. Papillary muscle distance and medio-lateral annular MV diameter were assessed in an midoesophageal two-chamber view.

*PLA = posterior mitral valve leaflet angle; ALA = anterior mitral valve leaflet angle; MV = mitral valve*
