# Supplementary material for: Impact of asymmetric tethering on outcomes after edge-to-edge mitral valve repair for secondary mitral regurgitation
Source: Clin Res Cardiol. 2021 Nov 16;111(8):869–80. doi: 10.1007/s00392-021-01961-5 (PMC9334427; doi:10.1007/s00392-021-01961-5)
Supplement: Supplementary file 2 — Supplementary file2 (DOCX 59 KB) [file 392_2021_1961_MOESM2_ESM.docx]

**SUPPLEMENTARY MATERIAL:**

| **Supplementary Table 1: Clinical and echocardiographic characteristics by tethering symmetry** | | | | | | | |
| --- | --- | --- | --- | --- | --- | --- | --- |
| **Parameter** | **Overall study cohort** | **Asymmetric postero-anterior tethering** | **Symmetric postero-anterior tethering** | **p-value** | **Asymmetric medio-lateral tethering** | **Symmetric**  **medio-lateral tethering** | **p-value** |
| Age, years | 71.6 ± 10.9 | 71.4 ± 10.7 | 71.6 ± 11.1 | 0.812 | 73.0 ± 11.7 | 71.1 ± 10.7 | 0.087 |
| Male Sex | 109 (60.9) | 42 (62.7) | 67 (60.4) | 0.758 | 29 (59.2) | 79 (61.2) | 0.802 |
| Previous MI | 61 (34.1) | 25 (37.3) | 35 (31.5) | 0.430 | 18 (36.7) | 42 (32.6) | 0.600 |
| Previous CABG | 25 (14.0) | 14 (20.9) | 11 (9.9) | 0.042 | 5 (10.2) | 20 (15.5) | 0.365 |
| Previous stroke or TIA | 18 (10.1) | 6 (9.0) | 11 (9.9) | 0.834 | 4 (8.2) | 14 (10.9) | 0.596 |
| ICD/CRT/PM | 88 (49.2) | 30 (44.8) | 58 (52.3) | 0.335 | 19 (38.8) | 69 (53.3) | 0.080 |
| Extracardiac arteriopathy | 26 (14.5) | 7 (10.4) | 18 (16.2) | 0.258 | 7 (14.3) | 19 (14.7) | 0.941 |
| Afib or flutter | 121 (67.6) | 48 (71.6) | 73 (65.8) | 0.417 | 36 (73.5) | 85 (65.9) | 0.334 |
| BMI, kg/m² | 25.4 ± 4.5 | 25.4 ± 5.1 | 25.5 ± 4.2 | 0.639 | 25.8 ± 4.9 | 25.3 ± 4.4 | 0.593 |
| EuroSCORE II | 8.3 ± 9.2 | 9.0 ± 9.3 | 8.0 ± 9.3 | 0.484 | 7.1 ± 5.4 | 8.8 ± 10.2 | 0.637 |
| Coronary artery disease | 106 (59.2) | 46 (68.7) | 59 (53.2) | 0.042 | 26 (53.1) | 79 (61.2) | 0.323 |
| eGFR, ml/min | 53.6 ± 22.8 | 52.5 ± 22.4 | 54.6 ± 23.0 | 0.376 | 50.9 ± 22.7 | 54.9 ± 22.6 | 0.143 |
| Previous TAVR or SAVR | 19 (10.6) | 7 (10.4) | 12 (10.8) | 0.940 | 6 (12.2) | 13 (10.1) | 0.677 |
| Medication  ACE/AT inhibitors  ß blocker  Calcium antagonists  Statins  ASA  Diuretics  Aldosterone antagonists | 123 (74.1)  140 (84.3)  12 (7.3)  93 (56.7)  94 (56.6)  154 (86.0)  85 (53.1) | 43 (72.9)  50 (84.7)  4 (6.8)  35 (60.3)  35 (59.3)  54 (91.5)  32 (56.1) | 79 (74.5)  89 (84.0)  8 (7.6)  58 (55.2)  57 (53.8)  99 (93.4)  53 (52.0) | 0.818  0.895  0.843  0.530  0.315  0.658  0.613 | 29 (67.4)  34 (79.1)  2 (4.8)  16 (39.0)  21 (48.8)  40 (93.0)  17 (42.5) | 94 (77.0)  106 (86.9)  9 (7.4)  76 (62.3)  71 (58.2)  113 (92.6)  68 (57.1) | 0.215  0.220  0.560  0.010  0.521  0.931  0.109 |

| TR Severity  0+  1+  2+  3+  4+ | 6 (3.4)  83 (46.9)  64 (36.0)  23 (12.9)  1 (0.6) | 3 (4.5)  36 (54.5)  20 (30.3)  7 (10.6)  0 (0.0) | 3 (2.7)  47 (42.3)  44 (39.6)  16 (14.4)  1 (0.9) | 0.436 | 0 (0.0)  16 (33.3)  26 (54.2)  6 (12.5)  0 (0.0) | 6 (4.7)  67 (51.9)  38 (29.5)  17 (13.2)  1 (0.8) | 0.025 |
| --- | --- | --- | --- | --- | --- | --- | --- |
| MR EROA PISA, cm² | 0.26 ± 0.15 | 0.28 ± 0.18 | 0.24 ±0.12 | 0.275 | 0.28 ± 0.19 | 0.25 ± 0.13 | 0.562 |
| MR RegVol PISA, ml | 37.4 ± 19.3 | 39.1 ± 19.8 | 36.1 ± 19.0 | 0.366 | 38.5 ± 19.9 | 36.6 ± 18.9 | 0.668 |
| MR vena contracta, cm | 0.69 ± 0.22 | 0.72 ± 0.22 | 0.68 ± 0.20 | 0.236 | 0.69 ± 0.21 | 0.69 ± 0.20 | 0.813 |
| LV-EDV, ml | 178.9 ± 69.9 | 190.5 ± 84.3 | 172.6 ± 59.0 | 0.279 | 178.8 ± 62.4 | 178.9 ± 72.6 | 0.756 |
| LV-ESV, ml | 117.0 ± 57.8 | 124.4 ± 66.8 | 113.2 ± 51.6 | 0.406 | 117.1 ± 53.8 | 117.0 ± 59.3 | 0.872 |
| LV-EDD, mm | 61.1 ± 10.5 | 61.4 ± 11.1 | 60.9 ± 10.2 | 0.964 | 61.1 ± 8.9 | 61.1 ± 11.0 | 0.671 |
| LV-ESD, mm | 52.3 ± 10.6 | 53.0 ± 11.1 | 52.0 ± 10.3 | 0.777 | 53.3 ± 9.9 | 51.9 ± 10.8 | 0.225 |
| LV-EF, % | 35.3 ± 11.2 | 35.6 ± 11.6 | 35.1 ± 10.9 | 0.876 | 35.6 ± 9.9 | 35.2 ± 11.6 | 0.720 |
| LV length | 83.8 ± 12.3 | 83.6 ± 14.1 | 84.0 ± 11.2 | 0.861 | 83.3 ± 11.3 | 83.9 ± 12.7 | 0.742 |
| LV width | 57.3 ± 11.0 | 57.9 ± 12.1 | 57.1 ± 10.3 | 0.644 | 57.0 ± 10.8 | 57.5 ± 11.1 | 0.912 |
| LV-sphericity | 1.5 ± 0.2 | 1.5 ± 0.2 | 1.5 ± 0.2 | 0.279 | 1.5 ± 0.2 | 1.5 ± 0.2 | 0.898 |
| LA volume, ml | 113.6 ± 53.4 | 114.5 ± 51.7 | 113.4 ± 54.8 | 0.600 | 121.7 ± 52.1 | 110.7 ± 53.7 | 0.143 |
| Papillary muscle distance, mm | 28.7 ± 6.8 | 29.2 ± 7.8 | 28.5 ± 6.1 | 0.741 | 29.3 ± 7.1 | 28.5 ± 6.6 | 0.498 |
| ML MV annular diameter, mm | 38.7 ± 5.3 | 37.7 ± 4.9 | 39.4 ± 5.5 | 0.034 | 38.7 ± 5.3 | 38.6 ± 5.3 | 0.868 |
| ALA, °  S1  S2  S3 | 32.3 ± 11.3  33.8 ± 11.1  28.5 ± 10.7 | 31.3 ± 12.0  28.1 ± 8.6  26.9 ± 10.3 | 33.1 ± 10.8  37.2 ± 11.0  29.5 ± 11.0 | 0.215  <0.001  0.111 | 25.0 ± 10.5  22.9 ± 6.4  32.0 ± 8.6 | 35.1 ± 10.4  33.7 ± 11.1  27.1 ± 11.1 | <0.001  0.723  0.004 |

| PLA, °  S1  S2  S3 | 36.6 ± 24.9  48.2 ± 33.6  38.0 ± 33.0 | 38.2 ± 14.7  63.2 ± 48.7  37.6 ± 15.9 | 35.7 ± 29.6  39.2 ± 13.3  38.1 ± 40.1 | 0.031  <0.001  0.127 | 28.0 ± 10.9  49.9 ± 42.5  38.0 ± 13.3 | 40.1 ± 27.9  47.5 ± 30.0  37.9 ± 38.1 | <0.001  0.900  0.048 |
| --- | --- | --- | --- | --- | --- | --- | --- |
| Postero-anterior tethering symmetry  S1  S2  S3 | 1.24 ± 0.84  1.54 ± 1.22  1.46 ± 1.09 | 1.40 ± 0.62  2.33 ± 1.68  1.55 ± 0.77 | 1.17 ± 0.95  1.07 ± 0.29  1.41 ± 1.25 | 0.001  <0.001  0.003 | 1.38 ± 1.11  1.53 ± 0.98  1.22 ± 0.39 | 1.19 ± 0.17  1.55 ± 1.31  1.56 ± 1.25 | 0.439  0.980  0.445 |
| Medio-lateral tethering symmetry  S3-S1  S3-S2  S2-S1 | 1.31 ± 1.35  0.72 ± 0.32  1.91 ± 1.79 | 1.34 ± 1.85  0.72 ± 0.39  1.92 ± 1.88 | 1.26 ± 0.90  0.71 ± 0.26  1.90 ± 1.74 | 0.628  0.558  0.829 | 2.58 ± 2.82  0.88 ± 0.38  3.44 ± 2.82 | 0.82 ± 0.32  0.65 ± 0.26  1.33 ± 0.54 | <0.001  <0.001  <0.001 |
| AP MV annular diameter, mm  S1  S2  S3 | 36.6 ± 6.2  41.3 ± 6.5  37.3 ± 6.0 | 35.9 ± 5.7  41.0 ± 5.2  36.4 ± 5.4 | 37.1 ± 6.5  41.5 ± 7.1  37.9 ± 10.1 | 0.208  0.488  0.066 | 33.8 ± 6.0  40.2 ± 7.4  38.4 ± 6.1 | 37.8 ± 5.9  41.7 ± 6.1  36.9 ± 6.0 | <0.001  0.642  0.116 |
| MV annular sphericity  S1  S2  S3 | 1.08 ± 0.19  0.96 ± 0.36  1.06 ± 0.34 | 1.07 ± 0.19  0.91 ± 0.16  1.03 ± 0.20 | 1.08 ± 0.19  1.00 ± 0.44  1.08 ± 0.40 | 0.816  0.104  0.906 | 1.17 ± 0.19  1.03 ± 0.66  1.00 ± 0.21 | 1.04 ± 0.17  0.94 ± 0.14  1.08 ± 0.38 | <0.001  0.868  0.038 |
| AML length, mm  S1  S2  S3 | 26.7 ± 6.9  34.0 ± 6.5  27.1 ± 6.0 | 27.0 ± 6.9  34.5 ± 6.8  26.8 ± 6.7 | 26.5 ± 7.0  33.7 ± 6.3  27.3 ± 5.5 | 0.712  0.632  0.321 | 22.9 ± 6.4  33.3 ± 7.3  27.0 ± 5.9 | 28.1 ± 6.6  34.2 ± 6.2  27.1 ± 6.0 | <0.001  0.439  0.788 |
| PML length, mm  S1  S2  S3 | 15.9 ± 4.8  16.5 ± 4.4  16.3 ± 4.6 | 14.8 ± 4.6  15.9 ± 4.8  15.7 ± 4.2 | 16.6 ± 4.8  16.8 ± 4.2  16.7 ± 4.9 | 0.002  0.116  0.205 | 14.7 ± 5.0  16.3 ± 4.5  17.1 ± 5.7 | 16.4 ± 4.6  16.5 ± 4.5  16.0 ± 4.2 | 0.013  0.873  0.274 |

| Tenting height, mm  S1  S2  S3 | 6.5 ± 2.7  8.6 ± 3.0  6.5 ± 2.6 | 6.5 ± 2.7  8.9 ± 3.3  6.7 ± 3.0 | 6.5 ± 2.7  8.4 ± 2.9  6.5 ± 2.3 | 0.884  0.335  0.916 | 4.6 ± 1.8  8.0 ± 2.7  7.1 ± 2.2 | 7.2 ± 2.6  8.8 ± 3.1  6.3 ± 2.7 | <0.001  0.182  0.020 |
| --- | --- | --- | --- | --- | --- | --- | --- |
| Tenting area, mm²  S1  S2  S3 | 185 ± 102  270 ± 113  182 ± 92 | 183.5 ± 104.5  265.7 ± 123.5  177.6 ± 103.2 | 186.9 ± 100.1  272.1 ± 107.2  184.6 ± 84.7 | 0.771  0.716  0.307 | 101.5 ± 55.8  265.9 ± 109.1  210.6 ± 83.2 | 217.6 ± 96.5  270.0 ± 115.0  170.0 ± 91.9 | <0.001  0.972  0.001 |
| MV mean PG, mmHg | 1.7 ± 0.8 | 1.9 ± 0.9 | 1.6 ± 0.8 | 0.024 | 1.6 ± 0.8 | 1.7 ± 0.9 | 0.576 |
| TAPSE, mm | 17.7 ± 4.6 | 17.6 ± 4.8 | 17.8 ± 4.5 | 0.716 | 18.6 ± 5.3 | 17.4 ± 4.3 | 0.225 |
| TR max PG, mmHg | 37.1 ± 12.7 | 37.9 ± 12.3 | 36.6 ± 13.0 | 0.589 | 35.7 ± 10.3 | 37.5 ± 13.4 | 0.544 |
| sPAP, mmHg | 44.4 ± 14.5 | 43.6 ± 14.4 | 44.9 ± 14.6 | 0.636 | 45.0 ± 12.9 | 44.2 ± 15.1 | 0.787 |
| ACE = angiotensin conversion enzyme; ALA = Anterior mitral valve leaflet angle; AML = anterior mitral valve leaflet; AP = postero-anterior; ASA = acetylsalicylic acid; AT = Angiotensin; BMI = Body mass index; CABG = Coronary artery bypass graft; CRT = Cardiac resynchronization therapy; eGFR = Estimated glomerular filtration rate; EROA = Effective regurgitant orifice area; HTX = Heart transplantation; ICD = Implantable cardioverter-defibrillator; LA = Left atrium; LV = Left ventricle; LV-EDD = Left ventricular end-diastolic dimension; LV-EDV = Left ventricular end-systolic volume; LV-EF = Left ventricular ejection fraction; LV-ESD = Left ventricular end-systolic dimension; LV-ESV = Left ventricular end-diastolic volume; MI = Myocardial infarction; ML = Mediolateral MR = Mitral regurgitation; MV = Mitral valve; NYHA = New York Heart Association; PG = Pressure gradient; PISA = Proximal isovelocity surface area; PLA = Posterior mitral valve leaflet angle; PML = posterior mitral valve leaflet; SAVR = Surgical aortic valve repair; ; sPAP = Systolic pulmonary artery pressure; TAPSE = Tricuspid annular plane systolic excursion; TAVR = Transcatheter aortic valve repair; TIA = Transient ischemic attack; TR = Tricuspid regurgitation; | | | | | | | |

| **Supplementary Table 2 – Logistic regression model for predicting asymmetric postero-anterior tethering** | | | | | | |
| --- | --- | --- | --- | --- | --- | --- |
|  | univariable | | | multivariable | | |
|  | HR | CI | p-value | HR | CI | p-value |
| MR EROA PISA, cm² | 6.147 | 0.727-52.0 | 0.096 |  |  |  |
| MR RegVol PISA, ml | 1.009 | 0.993-1.025 | 0.261 |  |  |  |
| MR vena contracta, cm | 2.759 | 0.611-12.47 | 0.178 |  |  |  |
| LV-EF, % | 1.005 | 0.978-1.033 | 0.716 |  |  |  |
| LV-EDV, ml | 1.004 | 0.999-1.008 | 0.114 |  |  |  |
| LV-ESV, ml | 1.003 | 0.998-1.009 | 0.229 |  |  |  |
| LV-EDD, mm | 1.004 | 0.974-1.035 | 0.779 |  |  |  |
| LV-ESD, mm | 1.009 | 0.979-1.040 | 0.573 |  |  |  |
| LA volume, ml | 1.000 | 0.995-1.006 | 0.893 |  |  |  |
| LV length, mm | 0.997 | 0.973-1.023 | 0.846 |  |  |  |
| LV width, mm | 1.007 | 0.979-1.036 | 0.638 |  |  |  |
| LV Sphericity index | 0.461 | 0.129-2.528 | 0.461 |  |  |  |
| Papillary muscle distance | 1.015 | 0.965-1.068 | 0.559 |  |  |  |
| MV mean PG, mmHg | **1.492** | **1.033-2.153** | **0.033** | **1.529** | **1.052-2.223** | **0.026** |
| TAPSE | 0.992 | 0.928-1.060 | 0.812 |  |  |  |
| sPAP, mmHg | 0.994 | 0.966-1.023 | 0.680 |  |  |  |
| TR max PG, mmHg | 1.008 | 0.982-1.034 | 0.558 |  |  |  |
| Age, years | 0.998 | 0.971-1.027 | 0.915 |  |  |  |
| Male Sex | 1.018 | 0.541-1.915 | 0.955 |  |  |  |
| BMI, kg/m² | 1.001 | 0.934-1.072 | 0.985 |  |  |  |
| eGFR, ml/min | 0.995 | 0.981-1.009 | 0.478 |  |  |  |
| Previous MI | 1.331 | 0.702-2.521 | 0.381 |  |  |  |
| Previous CABG | 2.377 | 1.009-5.603 | 0.048 |  |  |  |
| Prior TAVR or SAVR | 0.814 | 0.290-2.284 | 0.695 |  |  |  |
| Previous stroke or TIA | 0.885 | 0.311-2.516 | 0.819 |  |  |  |
| Atrial fibrillation or flutter | 1.280 | 0.660-2.483 | 0.464 |  |  |  |
| Ischemic heart disease | **1.964** | **1.038-3.716** | **0.038** | **2.190** | **1.134-4.228** | **0.020** |
| ICD/CRT | 0.727 | 0.395-1.338 | 0.305 |  |  |  |
| Extracardiac arteriopathy | 0.596 | 0.235-1.514 | 0.277 |  |  |  |
| NYHA functional class IV | 0.769 | 0.378-1.567 | 0.470 |  |  |  |
| MR Severity IV | 1.313 | 0.709-2.431 | 0.387 |  |  |  |
| TR Severity ≥3+ | 0.649 | 0.254-1.659 | 0.367 |  |  |  |
| n=175; ALA = Anterior mitral valve leaflet angle; ASA = acetylsalicylic acid; BMI = Body mass index; CABG = Coronary artery bypass graft; CI = confidence interval; eGFR = Estimated glomerular filtration rate; HR = hazard ratio; HTX = Heart transplantation; EROA = Effective regurgitant orifice area; LA = Left atrium; LV = Left ventricle; LV-EDD = Left ventricular end-diastolic dimension; LV-EDV = Left ventricular end-systolic volume; LV-EF = Left ventricular ejection fraction; LV-ESD = Left ventricular end-systolic dimension; LV-ESV = Left ventricular end-diastolic volume; MI = Myocardial infarction; MR = Mitral regurgitation; MV = Mitral valve; NYHA = New York Heart Association; PG = Pressure gradient; PISA = Proximal isovelocity surface area; PLA = Posterior mitral valve leaflet angle; SAVR = Surgical aortic valve repair; sPAP = Systolic pulmonary artery pressure; TAPSE = Tricuspid annular plane systolic excursion; TAVR = Transcatheter aortic valve repair; TIA = Transient ischemic attack TR = Tricuspid regurgitation; | | | | | | |

| **Supplementary Table 3: Procedural characteristics and NYHA follow-up by tethering symmetry** | | | | | | | |
| --- | --- | --- | --- | --- | --- | --- | --- |
| **Parameter** | **Overall study cohort** | **Asymmetric postero-anterior tethering** | **Symmetric postero-anterior tethering** | **p-value** | **Asymmetric medio-lateral tethering** | **Symmetric medio-lateral tethering** | **p-value** |
| MR Severity (n=178)  3+  4+ | 106 (59.6)  72 (40.4) | 37 (55.2)  30 (44.8) | 68 (61.3)  43 (38.7) | 0.428 | 30 (61.2)  19 (38.8) | 76 (58.9)  53 (41.1) | 0.779 |
| MR Severity postprocedural (n=178)  1+  2+  3+  4+ | 116 (65.7)  46 (25.8)  12 (7.3)  1 (1.1) | 37 (55.2)  20 (29.9)  8 (11.9)  2 (3.0) | 79 (71.8)  27 (24.5)  4 (3.6)  0 (0.0) | 0.021 | 33 (67.3)  12 (24.5)  4 (8.2)  0 (0.0) | 84 (65.1)  35 (27.1)  8 (6.2)  2 (1.6) | 0.781 |
| Implanted Clips (n=178)  0  1  2  3  4 | 11 (6.2)  80 (44.9)  82 (46.1)  4 (2.2)  1 (0.6) | 9 (13.4)  24 (35.8)  31 (46.3)  3 (4.5)  0 (0.0) | 1 (0.9)  55 (49.5)  51 (46.4)  2 (1.8)  1 (0.9) | 0.005 | 2 (4.1)  22 (44.9)  22 (44.9)  2 (4.1)  1 (2.0) | 8 (6.2)  58 (45.0)  60 (46.5)  3 (2.3)  0 (0.0) | 0.615 |
| NYHA functional class (n=178)  II  III  IV | 1 (0.6)  132 (74.2)  45 (25.3) | 0 (0.0)  52 (77.6)  15 (22.4) | 1 (0.9)  79 (71.2)  31 (27.9) | 0.513 | 0 (0.0)  35 (71.4)  14 (28.6) | 1 (0.8)  97 (75.2)  31 (24.0) | 0.692 |
| NYHA class follow-up (n=121)  I  II  III  IV | 19 (15.7)  57 (47.1)  38 (31.4)  7 (5.8) | 6 (15.8)  20 (52.6)  10 (26.3)  2 (5.3) | 13 (15.5)  38 (45.2)  28 (33.3)  5 (6.0) | 0.864 | 4 (14.3)  11 (39.3)  12 (42.9)  1 (3.6) | 15 (16.1)  46 (49.5)  26 (28.0)  6 (6.5) | 0.503 |
| Data are presented as number (%)  MR = mitral regurgitation; NYHA = New York Heart Association; ML = medio-lateral; AP = postero-anterior | | | | | | | |

| **Supplementary Table 4 – Logistic regression model for predicting asymmetric medio-lateral tethering** | | | | | | |
| --- | --- | --- | --- | --- | --- | --- |
|  | univariable | | | multivariable | | |
|  | HR | CI | p-value | HR | CI | p-value |
| MR EROA PISA, cm² | 3.714 | 0.439-31.45 | 0.229 |  |  |  |
| MR RegVol PISA, ml | 1.005 | 0.988-1.022 | 0.557 |  |  |  |
| MR vena contracta, cm | 0.851 | 0.163-4.455 | 0.849 |  |  |  |
| LV-EF, % | 1.004 | 0.974-1.035 | 0.806 |  |  |  |
| LV-EDV, ml | 1.000 | 0.995-1.005 | 0.991 |  |  |  |
| LV-ESV, ml | 1.000 | 0.994-1.006 | 0.989 |  |  |  |
| LV-EDD, mm | 1.000 | 0.967-1.035 | 0.981 |  |  |  |
| LV-ESD, mm | 1.014 | 0.981-1.048 | 0.408 |  |  |  |
| LA volume, ml | 1.004 | 0.997-1.010 | 0.243 |  |  |  |
| LV length, mm | 0.778 | 0.969-1.024 | 0.778 |  |  |  |
| LV width, mm | 0.996 | 0.966-1.027 | 0.788 |  |  |  |
| LV Sphericity index | 1.001 | 0.205-4.889 | 0.999 |  |  |  |
| Papillary muscle distance | 1.018 | 0.964-1.075 | 0.528 |  |  |  |
| MV mean PG, mmHg | 0.894 | 0.597-1.339 | 0.588 |  |  |  |
| TAPSE | 1.055 | 0.983-1.132 | 0.136 |  |  |  |
| sPAP, mmHg | 1.004 | 0.973-1.035 | 0.808 |  |  |  |
| TR max PG, mmHg | 0.988 | 0.961-1.017 | 0.427 |  |  |  |
| Age, years | 1.018 | 0.985-1.051 | 0.290 |  |  |  |
| Male Sex | 0.918 | 0.469-1.795 | 0.802 |  |  |  |
| BMI, kg/m² | 1.028 | 0.956-1.104 | 0.459 |  |  |  |
| eGFR, ml/min | 0.992 | 0.977-1.007 | 0.304 |  |  |  |
| Previous MI | 1.203 | 0.605-2.392 | 0.599 |  |  |  |
| Previous CABG | 0.619 | 0.219-1.753 | 0.367 |  |  |  |
| Prior TAVR or SAVR | 1.245 | 0.445-3.483 | 0.676 |  |  |  |
| Previous stroke or TIA | 0.730 | 0.228-2.337 | 0.596 |  |  |  |
| Atrial fibrillation or flutter | 1.433 | 0.690-2.978 | 0.334 |  |  |  |
| Ischemic heart disease | 0.715 | 0.368-1.389 | 0.323 |  |  |  |
| ICD/CRT | 0.551 | 0.282-1.077 | 0.081 |  |  |  |
| Extracardiac arteriopathy | 0.965 | 0.378-2.462 | 0.940 |  |  |  |
| NYHA functional class IV | 1.265 | 0.603-2.650 | 0.534 |  |  |  |
| MR Severity IV | 0.908 | 0.463-1.781 | 0.779 |  |  |  |
| TR Severity ≥3+ | 0.881 | 0.327-2.370 | 0.802 |  |  |  |
| n=178; ALA = Anterior mitral valve leaflet angle; ASA = acetylsalicylic acid; BMI = Body mass index; CABG = Coronary artery bypass graft; CI = confidence interval; eGFR = Estimated glomerular filtration rate; HR = hazard ratio; HTX = Heart transplantation; EROA = Effective regurgitant orifice area; LA = Left atrium; LV = Left ventricle; LV-EDD = Left ventricular end-diastolic dimension; LV-EDV = Left ventricular end-systolic volume; LV-EF = Left ventricular ejection fraction; LV-ESD = Left ventricular end-systolic dimension; LV-ESV = Left ventricular end-diastolic volume; MI = Myocardial infarction; MR = Mitral regurgitation; MV = Mitral valve; NYHA = New York Heart Association; PG = Pressure gradient; PISA = Proximal isovelocity surface area; PLA = Posterior mitral valve leaflet angle; SAVR = Surgical aortic valve repair; sPAP = Systolic pulmonary artery pressure; TAPSE = Tricuspid annular plane systolic excursion; TAVR = Transcatheter aortic valve repair; TIA = Transient ischemic attack TR = Tricuspid regurgitation; | | | | | | |

| **Supplementary Table 5 – COX regression model for all-cause two-year mortality (uni- and multivariable)** | | | | | | |
| --- | --- | --- | --- | --- | --- | --- |
|  | univariable | | | multivariable | | |
|  | HR | CI | p-value | HR | CI | p-value |
| Asymmetric medio-lateral tethering | **2.609** | **1.459-4.665** | **0.001** | **2.901** | **1.543-5.451** | **0.001** |
| Asymmetric postero-anterior tethering | **2.332** | **1.305-4.169** | **0.004** | **2.769** | **1.426-5.376** | **0.003** |
| MR EROA PISA, cm² | 1.408 | 0.186-10.67 | 0.741 |  |  |  |
| MR RegVol PISA, ml | 0.989 | 0.973-1.005 | 0.167 |  |  |  |
| MR vena contracta, cm | 1.412 | 0.382-5.215 | 0.605 |  |  |  |
| LV-EF, per 10% decrease | **1.336** | **1.006-1.775** | **0.045** | **1.417** | **1.037-1.937** | **0.029** |
| LV-EDV, ml | 1.002 | 0.998-1.006 | 0.329 |  |  |  |
| LV-ESV, ml | 1.004 | 1.000-1.009 | 0.064 |  |  |  |
| LV-EDD, mm | 0.993 | 0.965-1.022 | 0.642 |  |  |  |
| LV-ESD, mm | 1.005 | 0.978-1.032 | 0.741 |  |  |  |
| LA volume, ml | 1.002 | 0.997-1.008 | 0.382 |  |  |  |
| LV length, mm | 1.018 | 0.994-1.043 | 0.139 |  |  |  |
| LV width, mm | 1.010 | 0.984-1.037 | 0.436 |  |  |  |
| LV Sphericity index | 1.671 | 0.464-6.015 | 0.432 |  |  |  |
| Papillary muscle distance | 1.025 | 0.980-1.072 | 0.287 |  |  |  |
| MV mean PG, mmHg | 0.814 | 0.555-1.193 | 0.292 |  |  |  |
| TAPSE | 0.954 | 0.892-1.021 | 0.174 |  |  |  |
| sPAP, mmHg | 0.971 | 0.975-1.027 | 0.971 |  |  |  |
| TR max PG, mmHg | 1.007 | 0.983-1.031 | 0.593 |  |  |  |
| Age, years | 1.012 | 0.984-1.042 | 0.393 |  |  |  |
| Male Sex | 1.422 | 0.778-2.602 | 0.253 |  |  |  |
| BMI, kg/m² | 0.981 | 0.920-1.045 | 0.548 |  |  |  |
| eGFR, 10 ml/min decrease | **1.265** | **1.086-1.478** | **0.002** | **1.247** | **1.058-1.471** | **0.008** |
| Previous MI | 1.727 | 0.971-3.072 | 0.063 |  |  |  |
| Previous CABG | **2.637** | **1.367-5.089** | **0.004** | **2.298** | **1.129-4.676** | **0.022** |
| Prior TAVR or SAVR | 1.278 | 0.572-2.853 | 0.550 |  |  |  |
| Previous stroke or TIA | 1.246 | 0.493-3.149 | 0.642 |  |  |  |
| Atrial fibrillation or flutter | 1.792 | 0.891-3.605 | 0.102 |  |  |  |
| Ischemic heart disease | 1.235 | 0.681-2.240 | 0.487 |  |  |  |
| ICD/CRT | 1.607 | 0.898-2.878 | 0.110 |  |  |  |
| Extracardiac arteriopathy | **3.096** | **1.631-5.874** | **0.001** | **2.627** | **1.194-5.781** | **0.016** |
| Postprocedural MR severity ≥3+ | 1.645 | 0.650-4.164 | 0.293 |  |  |  |
| NYHA functional class IV | 1.371 | 0.734-2.563 | 0.323 |  |  |  |
| MR Severity IV | 1.005 | 0.563-1.791 | 0.988 |  |  |  |
| TR Severity ≥3+ | 1.261 | 0.589-2.699 | 0.551 |  |  |  |
| n=164; ALA = Anterior mitral valve leaflet angle; ASA = acetylsalicylic acid; BMI = Body mass index; CABG = Coronary artery bypass graft; CI = confidence interval; eGFR = Estimated glomerular filtration rate; HR = hazard ratio; HTX = Heart transplantation; EROA = Effective regurgitant orifice area; LA = Left atrium; LV = Left ventricle; LV-EDD = Left ventricular end-diastolic dimension; LV-EDV = Left ventricular end-systolic volume; LV-EF = Left ventricular ejection fraction; LV-ESD = Left ventricular end-systolic dimension; LV-ESV = Left ventricular end-diastolic volume; MI = Myocardial infarction; MR = Mitral regurgitation; MV = Mitral valve; NYHA = New York Heart Association; PG = Pressure gradient; PISA = Proximal isovelocity surface area; PLA = Posterior mitral valve leaflet angle; SAVR = Surgical aortic valve repair; sPAP = Systolic pulmonary artery pressure; TAPSE = Tricuspid annular plane systolic excursion; TAVR = Transcatheter aortic valve repair; TIA = Transient ischemic attack TR = Tricuspid regurgitation; | | | | | | |
